# Macrolide-lincosamide-streptogramin resistance phenotypes and genotypes of coagulase-positive *Staphylococcus aureus* and coagulase-negative staphylococcal isolates from bovine mastitis

**DOI:** 10.1186/s12917-015-0492-8

**Published:** 2015-07-25

**Authors:** Longping Li, Weiwei Feng, Zhiping Zhang, Huping Xue, Xin Zhao

**Affiliations:** College of Animal Science and Technology, Northwest A&F University, Yang Ling, Shaanxi P. R. China; Department of Animal Science, McGill University, 21,111 Lakeshore, Ste. Anne de Bellevue, H9X 3 V9 QC Canada

**Keywords:** Macrolide resistance, Disk-diffusion induction test, Staphylococci, Bovine milk

## Abstract

**Background:**

There are limited data available on macrolide-lincosamide-streptogramin (MLS) resistance of *Staphylococcus aureus* (*S. aureus*) and coagulase-negative staphylococci (CoNS) from bovine milk in China. To address this knowledge gap, MLS resistance was determined in 121 *S. aureus* and 97 CoNS isolates. Minimum inhibitory concentrations (MICs) of MLS antibiotics were determined by an agar dilution method, while differentiation of MLS phenotypes was performed by a double-disc diffusion test. MLS resistance genotypes were determined by PCR for corresponding resistance genes.

**Results:**

Forty (33.1 %) *S. aureus* and 65 (67.0 %) CoNS were resistant to erythromycin, whereas all 218 isolates were susceptible to quinupristin/dalfopristin. Among 40 erythromycin-resistant (ER-R) *S. aureus* and 65 ER-R CoNS isolates, 38 *S. aureus* and 40 CoNS isolates exhibited the inducible MLS (iMLS) resistance phenotype and 2 *S. aureus* and 20 CoNS isolates expressed the constitutive MLS resistance (cMLS) phenotype. At the same time, 5 CoNS isolates exhibited resistance to erythromycin but susceptibility to clindamycin (the MS phenotype). An inactivating enzyme gene *lnu(A)*, methylase genes *erm(C)* and *erm(B)*, efflux genes *msr(A)/msr(B)*, a phosphotransferase gene *mph(C)*, an esterase gene *ere(A)* and the streptogramin resistance determinant *vga(A)* were detected individually or in combinations. Among them, genes *lnu(A)*, *erm(C)* and *mph(C)* predominated. The *ereA* gene was detected for the first time in staphylococci of bovine milk origin. Resistance genes also existed in erythromycin-susceptible isolates.

**Conclusions:**

Our study demonstrated a high level of resistance to MLS antibiotics in staphylococci from bovine mastitic milk, especially with a high rate of the iMLS phenotype in *S. aureus* isolates. These data suggest that MLS antibiotics should be used judiciously to treat or prevent bovine mastitis caused by staphylococci.

## Background

Bovine mastitis is the most costly disease for the dairy industry worldwide. Although a wide variety of pathogens have been isolated as causative agents of this disease, *Staphylococcus aureus* (*S. aureus*) is considered as one of the most important pathogens due to its resistance to certain antibiotics and its propensity to recur chronically. Recently, coagulase-negative staphylococci (CoNS) have been considered as opportunistic pathogens that cause bovine mastitis in many countries and could be therefore described as emerging mastitis pathogens [1, 2]. Increasing attention has been paid to CoNS in both subclinical and clinical mastitis cases throughout the world [3, 4]. Macrolide-lincosamide-streptogramin (MLS) antibiotics, including erythromycin, clindamycin and spiramycin, are frequently used for treatment of bovine mastitis [5, 6]. Thus, results from an *in vitro* susceptibility testing are an important tool to guide a veterinarian in selecting the most efficacious antimicrobial agent(s) for therapeutic and prophylactic intervention.

Three mechanisms are mainly responsible for acquiring resistance to MLS antibiotics in staphylococci: (1) target site modifications by methylation or mutation; (2) active efflux of antibiotics; or (3) inactivation of antibiotics. The first mechanism includes target site modifications by a methylase encoded by one or more of the *erm* genes, methylating 23S rRNA and thereby altering binding sites for MLS antibiotics [7]. Phenotypically, this resistance appears either inducible (resistant to 14- and 15-membered macrolides and susceptible to 16-membered macrolides, lincosamides and streptogramin B) or constitutive (resistant to all forms of these antibiotics) [8]. The second mechanism involves a macrolide efflux pump encoded by *msr(A)* and/or *msr(B)* genes. This pump protein belongs to the ABC transporter family and exports 14-membered macrolides and streptogramin B antibiotics from bacterial cells, while lincosamide and streptogramin A antibiotics remain unaffected (the MS phenotype) [9]. The third mechanism encompasses several enzymes. A lincosamide nucleotidyltransferase encoded by the *lnu(A)* gene confers resistance only to lincosamides and has been detected in CoNS isolates from bovine mastitis [10]. Esterases encoded by *ere(A)/(B)* genes hydrolyze the lactone ring of the macrocyclic nucleus [11]. Furthermore, *vga(A)/(B)* genes have been characterized as a determinant of streptogramin A resistance [11]. Finally, the macrolide phosphotransferase C encoded by the *mph(C)* gene inactivates some macrolide antibiotics and has been detected in CoNS isolated from bovine subclinical mastitis [12].

The reported resistance of *S. aureus* and CoNS isolated from bovine mastitis to MLS antibiotics in different countries was generally low [12, 13]. Meanwhile, there was a paucity of data regarding MLS-resistance phenotypes and genotypes of *S. aureus* and CoNS isolated from bovine mastitis in China, except one study [5]. The objective of this study was to determine the MLS resistance phenotypes and genotypes of 121 *S. aureus* and 97 CoNS isolates from mastitic milk from dairy farms of the Shaanxi province in Northwestern China.

## Methods

### *Bacterial* isolates

Milk samples were obtained from dairy cows with clinical mastitis under the ethical approval granted by the College of Animal Science and Technology, the Northwest Agriculture and Forestry (A&F) University (Permit Number: NWAFU1008), as described previously [14]. A total of 121 coagulase-positive *S. aureus* (CoPSA) and 97 coagulase-negative staphylococci (CoNS) were used in this study. CoNS species were identified by sequencing analyses based on *sodA* and/or *gap* genes [14]. Ninety-seven CoNS isolates encompassed 9 CoNS species: *Staphylococcus haemolyticus* (*S. haemolyticus,* n = 31), *Staphylococcus aureus* (*S. aureus,* n = 28), *Staphylococcus chromogenes* (*S. chromogenes,* n = 11), *Staphylococcus sciuri* (*S. sciuri,* n = 9), *Staphylococcus epidermidis* (*S. epidermidis,* n = 7), *Staphylococcus simulans* (*S. simulans,* n = 5), *Staphylococcus hyicus* (*S. hyicus,* n = 3), *Staphylococcus warneri* (*S. warneri,* n = 2) and *Staphylococcus saprophyticus* (*S. saprophyticus*, n = 1).

### Antibiotic susceptibility testing

Minimum inhibitory concentrations (MICs) of antimicrobial agents, including 14-membered (erythromycin), 15-membered (azithromycin), 13- and 15-membered mixture (tulathromycin) and 16-membered (tylosin and spiramycin) macrolides, lincosamides (clindamycin) and streptogramins (quinupristin/dalfopristin) were determined using an agar dilution method. Clindamycin, azithromycin, tulathromycin and spiramycin were purchased from Dalian Meilun Biology Technology Co., Ltd. (Dalian, China). Tylosin was purchased from Shanghai Kai Yang Biotechnology Co., Ltd. (Shanghai, China). Erythromycin was bought from Sigma-Aldrich (Beijing, China) and quinupristin/dalfopristin was obtained from Santa Cruz biotechnology, Inc. (Shanghai, China). The approved veterinary specific CLSI MIC breakpoints of erythromycin and clindamycin are ≥ 8 μg/mL and 4 μg/mL, respectively [15]. MIC breakpoint for spiramycin (≥32 μg/mL) was adopted from the Swedish Antibiotic Utilisation and Resistance in Human Medicine (SWEDRES) and Swedish Veterinary Antimicrobial Resistance Monitoring (SVARM) system (SWEDRES-SVARM) [16]. The MIC breakpoint for tylosin (≥20 μg/mL) was based on the Veterinary Antimicrobial Decision Support (VADS) according to a previous study [5]. There were no veterinary specific CLSI, VADS or SWEDRES-SVARM-approved breakpoints for azithromycin and quinupristin-dalfopristin. Therefore, we used a human specific CLSI document M100-S21 [17] as a reference to determine breakpoints for azithromycin (≥8 μg/mL) and quinupristin-dalfopristin (≥4 μg/mL). In addition, there was no CLSI, VADS or SVARM-approved breakpoint for tulathromycin. The standard reference strain *S. aureus* ATCC 29213 served as a quality control in every test run.

### Detection of MLS resistance phenotypes

In order to differentiate different types of resistance phenotype for erythromycin-resistant (ER-R) isolates, a double-disk diffusion test (D test) was performed with erythromycin (15 μg/disc) and clindamycin (2 μg/disc), following the procedure recommended by CLSI [17]. Staphylococcal isolates showing resistance to erythromycin (zone size ≤13 mm) but being sensitive to clindamycin (zone size ≥21 mm) and producing a D-shaped zone of inhibition around clindamycin with flattening towards erythromycin disc was defined as having an inducible type of MLS resistance (D^+^, iMLS). In addition, resistance to erythromycin (zone size ≤13 mm) as well as to clindamycin (zone size ≤14 mm) indicated a constitutive type of MLS resistance (cMLS). Staphylococcal isolates showing resistance to erythromycin (zone size ≤13 mm) while being sensitive to clindamycin (zone size ≥21 mm) with no blunting zone were classified as the MS phenotype.

### Detection of MLS resistance genotypes

Staphylococcal isolates were incubated in the Brain Heart Infusion broth (Oxoid) at 37 °C for 16-18 h. Then, bacteria were harvested by centrifugation. Plasmid and chromosome DNA of bacterial isolates were extracted using a commercial DNAout kit (Tiandz Inc., Beijing, China) as described previously [14]. The screening of MLS resistance determinants including methylase genes *erm(A), erm(B)* and *erm(C)*; phosphotransferase genes *mph(A)* and *mph(C)*; lincosamide nucleotidyltransferase genes *lnu(A)* and *lnu(B)*; erythromycin esterase genes *ere(A)* and *ere(B)*; streptogramin resistance genes *vga(A), vga(B), vgb(A)* and *vgb(B)*, and the macrolide efflux determinants *msr(A)/msr(B)* was performed by PCR using the specific primers as described in previous studies [11, 12, 18–20]. PCR products were randomly selected and sequenced to ensure specificity and accuracy. Sequence comparisons were performed using the Basic Local Alignment Search Tool (BLAST) program (http://www.ncbi.nlm.nih.gov/BLAST/).

## Results and discussion

### Characterization of MLS resistance phenotypes in erythromycin-resistant isolates and MIC distribution

Of the 121 coagulase-positive *S. aureus* (CoPSA) isolates, 40 were resistant to at least one MLS antibiotic. Thirty-eight out of 40 ER-R CoPSA (95 %) isolates exhibited iMLS phenotypes, whereas only 2 isolates expressed cMLS phenotypes, with no MS phenotype. The MICs of the macrolides and lincosamides antibiotics for the 40 ER-R CoPSA isolates are summarized in Table 1. Among ER-R CoPSA, 100 %, 100 %, 80 %, 17.5 % and 5 % of isolates were resistant to erythromycin, azithromycin, spiramycin, tylosin and clindamycin, respectively. The MIC_50_ and MIC_90_ values of 14-membered, 15-membered macrolides and 16-membered macrolide spiramycin were ≥128 μg/mL, while the MIC_50_ values of 16-membered macrolides tylosin and lincosamides were in the susceptible range. No isolate expressed resistance to quinupristin/dalfopristin (MICs ≤ 1 μg/mL).

**Table 1:** Minimum inhibitory concentrations (MICs) of 40 ER-R coagulase-positive *S. aureus* isolates

Antibiotic agents	The number of isolates inhibited in different MICs (μg mg^-1^)^a^												MIC50	MIC90
	≤0.125	0.25	0.5	1	2	4	8	16	32	64	128	≥256		
Erythromycin^b^	0	0	0	*0*	*0*	*0*	**0**	**1**	**3**	**2**	**6**	**28**	>256	>256
Clindamycin^b^	24	7	0	*1*	*6*	**0**	**0**	**0**	**0**	**0**	**0**	**2**	0.125	2
Spiramycin^c^	0	0	0	0	0	0	3	5	**0**	**2**	**30**	**0**	>128	>128
Tylosin^d^	0	0	3	5	25	0	*0*	*0*	**2**	**2**	**2**	**1**	2	64
Azithromycin^b^	0	0	0	0	0	0	**0**	**0**	**0**	**4**	**0**	**36**	>256	>256
Quinupristin-dalfopristin^b^	23	11	6	0	0	**0**	**0**	**0**	**0**	**0**	**0**	**0**	0.125	0.25
Tulathromycin^e^	0	0	0	0	0	0	0	0	0	0	5	35	>256	>256

^a^Susceptibility, intermediate resistance and resistance are shown in normal, italic and boldface fonts, respectively

^b^MIC breakpoints for erythromycin, azithromycin, clindamycin and quinupristin-dalfopristin were based on Clinical and Laboratory Standards Institute (CLSI) documents (CLSI, 2004, 2011)

^c^MIC breakpoints of spiramycin were based on the SWEDRES-SVARM (2012)

^d^MIC breakpoints of tylosin were based on the VADS according to a previous study [5]

^e^MIC breakpoints of this antibiotic were not available

MIC_50_, the MIC for 50 % of the organisms

MIC_90_, the MIC for 90 % of the organisms

Among 97 CoNS isolates, 65 isolates exhibited MLS resistance phenotypes. Among them, 40 showed the iMLS phenotype and 20 expressed the cMLS phenotype, while 5 exhibited the MS phenotype. The MICs of the antimicrobial agents tested are summarized in Table 2. Eighteen isolates with cMLS phenotypes exhibited a high-level of resistance to erythromycin, clindamycin, azithromycin, spiramycin and tylosin with MIC values of ≥256 μg/mL. Furthermore, 1 *S. haemolyticus* with the iMLS phenotype exhibited MICs ≥256 μg/mL for erythromycin and azithromycin while MICs for spiramycin and tylosin were 64 μg/mL and 128 μg/mL, respectively. In addition, 39 CoNS isolates with the iMLS phenotype showed a complete cross-resistance to erythromycin and azithromycin with MICs of ≥256 μg/mL. However, MIC values of 16-membered macrolides tylosin (2-8 μg/mL) and spiramycin (2-16 μg/mL) were in the susceptible ranges.

**Table 2:** Minimum inhibitory concentrations (MICs) of 97 CoNS isolates

Test agent	Species	The number of isolates inhibited in different MICs (μg mg^-1^)^a^											
		0.125	0.25	0.5	1	2	4	8	16	32	64	128	≥256
Erythromycin^b^	*S. chromogenes*		**2**	**3**				**1**					**5**
	*S. sciuri*		**2**	**5**							**1**		**1**
	*S. warneri*									**1**	**1**		
	*S. haemolyticus*			**1**									**30**
	*S. epidermidis*					*1*							**6**
	*S. hyicus*												**3**
	*S. saprophyticus*											**1**	
	*S. simulans*					*1*							**4**
	*S. aureus*		**12**	**5**				**1**					**10**
Clindamycin^b^	*S. chromogenes*	**6**	**1**			*3*							**1**
	*S. sciuri*	**1**				*7*							**1**
	*S. warneri*	**2**											
	*S. haemolyticus*	**1**	**8**			*10*							**12**
	*S. epidermidis*	**1**	**1**			*4*							**1**
	*S. hyicus*												**3**
	*S. saprophyticus*					*1*							
	*S. simulans*					*3*							**2**
	*S. aureus*	**16**	**6**			*6*							
Spiramycin^c^	*S. chromogenes*					**2**	**3**	**3**	**2**				**1**
	*S. sciuri*					**1**	**5**	**2**					**1**
	*S. warneri*							**2**					
	*S. haemolyticus*					**3**	**1**	**13**	**1**				**13**
	*S. epidermidis*					**1**	**1**	**2**	**2**				**1**
	*S. hyicus*												**3**
	*S. saprophyticus*							**1**					
	*S. simulans*											**3**	**2**
	*S. aureus*					**3**	**5**	**17**	**3**				
Tylosin^d^	*S. chromogenes*				**1**	**7**		*2*					**1**
	*S. sciuri*					**6**		*2*			**1**		
	*S. warneri*					**1**		*1*					
	*S. haemolyticus*					**13**		*4*		**1**		**1**	**12**
	*S. epidermidis*		**1**			**1**		*4*					**1**
	*S. hyicus*												**3**
	*S. saprophyticus*							*1*					
	*S. simulans*					**2**			**1**				**2**
	*S. aureus*				**2**	**20**		*5*					**1**
azithromycin^b^	*S. chromogenes*	**1**	**1**							**3**		**1**	**5**
	*S. sciuri*	**2**								**5**	**1**		**1**
	*S. warneri*										**2**		
	*S. haemolyticus*						**1**						**30**
	*S. epidermidis*						**1**						**6**
	*S. hyicus*												**3**
	*S. saprophyticus*											**1**	
	*S. simulans*							**1**					**4**
	*S. aureus*	**6**	**4**					**2**		**6**			**10**
quinupristin-dalfopristin^b^	*S. chromogenes*		**1**	**5**	**4**	**1**							
	*S. sciuri*			**1**		**8**							
	*S. warneri*			**1**	**1**								
	*S. haemolyticus*			**5**	**23**	**3**							
	*S. epidermidis*			**6**		**1**							
	*S. hyicus*			**2**	**1**								
	*S. saprophyticus*			**1**									
	*S. simulans*				**5**								
	*S. aureus*	**1**	**1**	**7**	**18**	**1**							
Tulathromycin^e^	*S. chromogenes*						**3**		**3**		**1**	**1**	**3**
	*S. sciuri*						**2**		**6**				**1**
	*S. warneri*						**1**		**1**				
	*S. haemolyticus*								**1**		**8**	**2**	**20**
	*S. epidermidis*						**1**				**1**		**5**
	*S. hyicus*												**3**
	*S. saprophyticus*						**1**						
	*S. simulans*						**1**				**2**		**2**
	*S. aureus*						**12**		**5**	**1**	**3**	**3**	**4**

^a^Intermediate resistance and resistance are shown in italic and boldface fonts, respectively

^b^MIC breakpoints for erythromycin, azithromycin, clindamycin and quinupristin-dalfopristin were based on Clinical and Laboratory Standards Institute (CLSI) documents (CLSI, 2004, 2011)

^c^MIC breakpoints of spiramycin were based on the SWEDRES-SVARM (2012)

^d^MIC breakpoints of tylosin were based on the VADS according to a previous study [5]

^e^MIC breakpoints of this antibiotic were not available

The iMLS phenotype rate of ER-R *S. aureus* (38/40) and ER-R CoNS (40/65) isolates was much higher in this study than previous studies, underlining the importance of routine screening of bovine *S. aureus* and CoNS isolates for inducible resistance phenotypes. Wang *et al.* [5] reported that the inducible MLS resistance phenotype was detected in 38 out of 72 *S. aureus* isolates from cows with clinical mastitis in Inner Mongolia of China. In another study, only 3 isolates with the iMLS phenotype were found out of 22 ER-R CoNS in Germany [12]. The reason for the higher rate of the iMLS phenotype in our study is not clear.

### Characterization of MLS resistance genotypes in erythromycin-resistant isolates

Among 40 ER-R CoPSA isolates, the most dominant resistance gene was *erm(C)* (38/40), followed by the *mph(C)* (27/40), *erm(B)* (14/40), *ere(A)* (14/40) and *vga(A)* (10/40) (Table 3). The *msr(A)/msr(B)* genes were found in 6 isolates, which were all positive for *erm(C)* or *erm(B)* genes and displayed iMLS phenotypes (Table 4). Considering the 65 ER-R CoNS isolates, *msr(A)/msr(B)* genes were present in 51 isolates, *erm(C)* in 46, *erm(B)* in 23, *mph(C)* in 25, *vga(A)* in 23 and *ere(A)* in 9 isolates. At least one of the MLS resistance genes was detected in each ER-R isolate. The simultaneous presence of two or more MLS antibiotic resistance genes was also detected (Table 4). The simultaneous presence of two or more macrolide resistance genes in the same *S. aureus* or CoNS isolate is well-known and has been reported previously for *S. aureus* or CoNS isolates from bovine mastitis [5, 12, 21].

**Table 3:** MLS resistance phenotypes and genotypes in ER-R coagulase-positive *S. aureus* and ER-R CoNS isolates

Species	Total number	Erythromycin-resistant number	Phenotype						The number of isolates containing resistance genes						
			iMLS	%	cMLS	%	MS	%	*lnuA*	*ermB*	*ermC*	*msrA/B*	*mphC*	*ereA*	*vgaA*
*S. chromogenes*	11	6	5	83.3	1	16.7	0	0	6	2	3	3	1	1	2
*S. sciuri*	9	2	0	50	1	0	1	50	2	2	0	1	0	0	0
*S. warneri*	2	2	0	0	0	0	2	100	2	1	0	1	1	0	0
*S. haemolyticus*	31	30	18	62	12	40	0	0	30	11	22	26	14	4	12
*S. epidermidis*	7	6	5	83.3	1	16.7	0	0	6	2	6	7	1	1	1
*S. hyicus*	3	3	0	0	3	100	0	0	3	3	2	2	1	1	1
*S. saprophyticus*	1	1	0	0	0	0	1	100	1	0	1	1	1	0	1
*S. simulans*	5	4	2	50	2	50	0	0	4	0	2	2	1	0	4
CoN *S. aureus* ^*a*^	28	11	10	91	0	0	1	9	11	3	10	8	5	2	2
Total CoNS	97	65	40	61.5	20	30.8	5	7.7	65	23	46	51	25	9	23
CoP *S. aureus* ^*b*^	121	40	38	95	2	5	0	0	40	14	38	6	27	14	10

iMLS, inducible expression of clindamycin resistance

cMLS, constitutive expression of clindamycin resistance

MS, erythromycin-resistant, clindamycin-susceptible, no induction

CoN *S. aureus* ^*a*^, coagulase-negative *S. aureus*

CoP *S. aureus* ^*b*^, coagulase-positive *S. aureus*

**Table 4:** Combinations of MLS resistance genes in 40 ER-R CoPSA and 65 ER-R CoNS isolates

Species	Total number	Erythromycin-resistant number	*ermB + ermC*				*ermC + msrA/msrB*				*ermC + mphC*				*ermC+ msrA/msrB + mphC*			
			n	iMLS	cMLS	MS	n	iMLS	cMSL	MS	n	iMLS	cMLS	MS	n	iMLS	cMLS	MS
*S. chromogenes*	11	6	1	1			1	1			1	1						
*S. sciuri*	9	2																
*S. warneri*	2	2																
*S. haemolyticus*	31	30	11	5	6		19	12	7		12	8	4		11	5	6	
*S. epidermidis*	7	6	1		1		4	3	1		1	1			1	1		
*S. hyicus*	3	3	2		2		2		2						1	1		
*S. saprophyticus*	1	1					1			1	1			1	1			1
*S. simulans*	5	4					1		1		1		1		1		1	
CoN *S. aureus* ^*a*^	28	11	2	2			8	7		1	5	4		1	3	2		1
Total CoNS	97	65	17	8	9	0	36	23	11	2	21	14	5	2	18	9	7	2
CoP *S. aureus* ^*b*^	121	40	18	17	1	0	6	6	0	0	27	26	1	0	6	5	1	0

iMLS, inducible expression of clindamycin resistance

cMLS, constitutive expression of clindamycin resistance

MS, erythromycin-resistant, clindamycin-susceptible, no induction

CoN *S. aureus* ^*a*^, coagulase-negative *S. aureus*

CoP *S. aureus* ^*b*^, coagulase-positive *S. aureus*

### Correlation between the MIC values of MLS resistance phenotypes and phenotypes

The possible relationship between MLS resistance phenotypes and genotypes was also explored. Among the 40 ER-R CoPSA isolates, 8 isolates with iMLS phenotypes were sensitive to 16-membered macrolides spiramycin and 32 isolates with iMLS phenotypes were sensitive to tylosin. Those isolates were all *erm(B)* and/or *erm(C)* positive. As for the 65 ER-R CoNS isolates, 4 *erm(B)* and/or *erm(C)* positive isolates with the MS phenotype and 28 *erm(B)* and/or *erm(C)* positive isolates with the iMLS phenotype were sensitive to 16-membered macrolides spiramycin and tylosin, respectively. Furthermore, 1 *S. warneri* with the MS phenotype and 9 CoNS isolates with the iMLS phenotype were also sensitive to 16-membered macrolides spiramycin and tylosin. Those 10 isolates were negative for *erm* genes but positive for other MLS resistance genes, such as *msr(A)/(B)*, *mph(C), ere(A), lnu(A)* or *vga(A)*. In general, *erm*-carrying ER-R *S. aureus* and CoNS isolates with iMLS or MS phenotypes possessed a high degree of resistance to erythromycin, azithromycin and clindamycin (inducible), while having a low rate of resistance to 16-membered macrolides tylosin and/or spiramycin.

It has been reported that the lactone rings of 16- and 14-membered macrolides adopt distinctly diverse conformations, thereby enabling the former compounds to avoid steric hindrance with the nucleotide A2058 mutation in *E. coli* [22]. Such a mechanism may be also responsible for differential sensitivity to 16- and 14-membered macrolides in staphylococci. In addition, differential effects of 14 and 15-membered macrolides versus 16-membered macrolides on expression of *erm* genes could contribute to our results. Expression of *erm* genes can be either inducible or constitutive. Inducible *erm* genes expression is controlled at a post-transcriptional level, which involves a structure upstream from the *erm* gene composing of a leader peptide and a series of inverted repeats. Formation of different mRNA secondary structures in this regulatory region in the presence or absence of an inducer allows or prevents the translation of the *erm* gene transcripts [8]. Only 14- and 15-membered macrolide can induce *erm* expression, while 16-membered macrolides, lincosamides, or streptogramins are not able to induce *erm* gene expression [8]. However, why 10 CoNS without the *erm* gene were also sensitive to 16-membered macrolides spiramycin and tylosin in this study is unclear and will be further studied. In addition, previous studies have shown that *erm* gene expression can quickly and irreversibly switch from inducible expression to constitutive expression under selective pressure due to the structural alterations (sequence deletions of varying length, duplications and mutations), which then renders the respective staphylococcal isolate resistant to all 14-, 15-, and 16-membered macrolides, lincosamides and streptogramin B antibiotics [23–25]. Therefore, different conformational rearrangements in the mRNA structure or structural alterations (deletions, duplications or mutations) in the upstream regulatory region of *erm* genes could be one of the plausible reasons of our isolates and such resistance mechanism will be further studied.

Other resistance genes may also play a role in the sensitivity to 16-membered macrolides. For example, *msr(A)/msr(B)* genes in CoPSA isolates. These genes encode an inducible efflux pump which is an ABC transporter. 14- and 15-membered macrolides are inducers and substrates for the pump, while clindamycin is neither an inducer nor a substrate [8]. In our research, all 6 *msr(A)/msr(B)*-carrying CoPSA isolates were also *erm(B)* and/or *erm(C)* genes positive. These isolates had the iMLS phenotype and exhibited MICs of ≥256 μg/mL for erythromycin while susceptible to 16-membered macrolides spiramycin and/or tylosin. However, the situation is much complicated for 51 *msr(A)/msr(B)*-carrying CoNS isolates. Among them, 31 (17 *S. haemolyticus*, 7 *S. aureus*, 4 *S. epidermidis* and 3 *S. chromogenes*) had the iMLS phenotype, 15 (9 *S. haemolyticus*, 2 *S. simulans*, 2 *S. hyicus*, 1 *S. epidermidis* and 1 *S. chromogenes*) had the cMLS phenotype and 5 (2 *S. warneri*, 1 *S. sciuri*, 1 *S. aureus* and 1 *S. saprophyticus*) had the MS phenotype.

### Characterization of MLS resistance genotypes in erythromycin-susceptible isolates

Among 81 erythromycin-susceptible (ER-S) CoPSA isolates, 79 isolates were positive for *lnu(A)*, 69 for *erm(C)*, 47 for *mph(C)*, 66 for *erm(B)*, 64 for *msr(A)/(B)*, 20 for *ere(A)* and 10 for *vga(A)* genes (Table 5), but all were susceptible to the corresponding antibiotics (erythromycin, azithromycin, spiramycin, tylosin or clindamycin) in the antibiotic susceptibility testing, due to unknown reasons. As for 32 ER-S CoNS isolates, 7, 9, 20, 11, 7 and 9 CoNS isolates harbored *erm(B)*, *erm(C), msr(A)/(B)*, *mph(C)*, *ere(A)*, *vga(A) genes,* respectively (Table 5)*.* Furthermore, the lincosamide nucleotidyltransferase gene, *lnu(A)*, was detected in all ER-R *S. aureus,* ER-R CoNS isolates, ER-S CoNS and 79 ER-S CoPSA isolates (Table 3; Table 5). The presence of *lnu(A)* among staphylococcal isolates from bovine mastitis has been reported [5, 10, 12]. The *lnu(A)* gene is mainly carried by small rolling-circle plasmids and it mediates only a low-level of resistance to the lincosamide pirlimycin [10]. The *ere(A)* gene was detected for the first time in staphylococci of bovine milk origin. Our results are in agreement with previous studies which detected *erm(C), lnu(A)*, *mph(C)* or *erm(A)* genes in susceptible *S. aureus* or CoNS isolates [12, 26, 27]. When Martineau et al. [26] subcultured 4 erythromycin susceptible strains harboring the *erm(C)* gene with increasing concentration of the antibiotic, they found that those susceptible strains all become resistant. Thus, we need to be vigilant when we use MLS antibiotics on dairy farms.

**Table 5:** MLS resistance genes in 81 ER-S coagulase-positive *S. aureus* and 32 ER-S CoNS isolates

Species	Total number	Erythromycin-susceptible number	*ermB*		*ermC*		*msrA/B*		*mphC*		*ereA*		*lnuA*		*vgaA*	
			n	%	n	%	n	%	n	%	n	%	n	%	n	%
*S. chromogenes*	11	5	1	20	1	20			2	40	3	60	5	100		
*S. sciuri*	9	7	1	14.3	1	14.3	4	57.1	7	100	1	14.3	7	100		
*S. warneri*	2	0														
*S. haemolyticus*	31	1	1	100									1	100		
*S. epidermidis*	7	1			1	100	1	100			1	100	1	100		
*S. hyicus*	3	0														
*S. saprophyticus*	1	0														
*S. simulans*	5	1					1	100			1	100	1	100	1	100
CoN *S. aureus* ^*a*^	28	17	4	23.5	6	35.3	15	88.2	2	11.8	1	5.9	17	100	8	47.1
Total CoNS	97	32	7	21.9	9	28.1	20	62.5	11	34.4	7	21.9	32	100	9	28.1
CoP *S. aureus* ^*b*^	121	81	66	81.5	69	85.2	64	79	47	58	20	24.7	79	97.5	10	12.3

CoN *S. aureus* ^*a*^, coagulase-negative *S. aureus*

CoP *S. aureus* ^*b*^, coagulase-positive *S. aureus*

## Conclusions

In summary, a very high rate of iMLS (95 %, 38/40) phenotype of ER-R *S. aureus* and MLS resistance phenotype (67 %, 65/97) of CoNS isolates from milk of mastitic cows was found in this study in comparison with previous studies, presumably due to extensive use of MLS antibiotics in dairy cows in our region. Our results suggest that MLS antibiotics should be used judiciously for therapeutic and prophylactic intervention of staphylococci infection.

## Abbreviations

cMLS: constitutive MLS resistance phenotype
CoNS: Coagulase-negative staphylococci
CoPSA: coagulase-positive *Staphylococcus aureus*
ER-R: erythromycin-resistant
ER-S: erythromycin-susceptible.
iMLS: inducible MLS resistance phenotype
MIC: Minimal inhibitory concentration
MLS: macrolide-lincosamide-streptogramin
